# A Lectin Disrupts Vector Transmission of a Grapevine Ampelovirus

**DOI:** 10.3390/v12080843

**Published:** 2020-08-01

**Authors:** Cecilia A. Prator, Rodrigo P. P. Almeida

**Affiliations:** Department of Environmental Science, Policy and Management, University of California, Berkeley, CA 94720, USA; cprator@berkeley.edu

**Keywords:** Grapevine leafroll-associated virus 3, Grapevine leafroll disease, Closteroviridae, *Ampelovirus*, *Planococcus ficus*, vine mealybug

## Abstract

Grapevine leafroll disease is one of the most important virus diseases of grapevines and occurs in every major grape-growing region of the world. The vector-transmission mechanisms of the causative agent, *Grapevine leafroll-associated virus 3* (GLRaV-3), remain poorly understood. We show that the vine mealybug, *Planococcus ficus*, feeds through a membrane feeding system on GLRaV-3 viral purifications from both *V. vinifera* and *N. benthamiana* and transmits the virus to test plants from plants from both species. Building on this strategy, we used an immunofluorescence approach to localize virions to two retention sites in *P. ficus* mouthparts. Assays testing molecules capable of blocking virus transmission demonstrated that GLRaV-3-transmission by *P. ficus* could be disrupted. Our results indicate that our membrane feeding system and transmission-blocking assays are a valid approach and can be used to screen other candidate blocking molecules.

## 1. Introduction

Insect vectors play an essential role in the dissemination of many viruses that cause diseases in humans, animals, and plants. Vector-transmission is a complex event in the virus disease cycle and can be divided into virus acquisition, retention, and inoculation processes. Most plant viruses rely on vectors for efficient transmission, such as fungi, nematodes, and arthropods [1]. Insects are the most common vectors of plant pathogens, and specific relationships, as well as specific transmission mechanisms, exist between a given virus genus and vector species. However, the molecular determinants of virus–vector interactions of many important viruses remain poorly understood.

Plant virus–insect vector relationships are characterized by properties associated with virus acquisition, retention, and transmission. There are four basic types of virus–vector relationships: nonpersistent and semipersistent, both of which are noncirculative, as well as circulative nonpropagative and propagative, where the virus enters via the gut and circulates or replicates within the insect body, respectively [2]. Specific viral proteins, molecular domains, and sequence motifs required for virus binding to vectors have been identified for the major viral genera transmitted by insect vectors [2]. Viral coat proteins and other accessory proteins (helper components) mediate interactions required for virus transmission [1]. The identity of putative virus receptors in vectors remains elusive. However, a recent study identified a protein-rich region (called “the acrostyle”) at the tip of aphid stylets, which functions as the binding site for *Cauliflower mosaic virus* (CaMV) [3]. This research was the first to identify proteins as putative vector receptors for a noncirculative plant virus as well as provided definitive proof of a noncirculative virus retention site located at the stylet tips of hemipteran insects [4]. Further advances in this system have allowed the identification of Stylin-1, the first cuticular protein specifically described in aphid stylets [5]. For other vector-borne plant viruses transmitted non-persistently, virus-encoded proteins specifically interact with receptors in the vector, allowing for retention and, consequently, successful transmission between plants [1]. In contrast to CaMV, the viral retention site for a semipersistent virus, *Lettuce infectious yellows virus* (LIYV), has been localized to the cibarium of whitefly vectors instead of stylet tips [6]. *Citrus tristeza virus* (CTV), another related semipersistent virus, has also been shown to be localized to the cibarium of an aphid vector [7].

*Grapevine leafroll-associated virus 3* (GLRaV-3) is associated with grapevine leafroll disease (GLD), an important problem in California and all grape-growing regions of the world [8]. GLRaV-3 is an 18Kb ssRNA *Ampelovirus* in the family Closteroviridae transmitted by phloem-sap-sucking mealybugs (Hemiptera, Pseudococcidae) and soft scales (Coccidae). GLRaV-3 is transmitted in a semipersistent manner; insect vectors can retain the virus for a period of a few days [9]. The specific retention site and transmission mechanisms for any *Ampelovirus* have not yet been described.

The vine mealybug (*Planococcus ficus*) is an invasive pest in most grape-growing regions worldwide, as well as an efficient vector of GLRaV-3 [10]. Mealybug nymphs and adult females have specialized piercing-sucking mouthparts that play an important role in virus transmission. Adult males have modified non-functional mouthparts and therefore are unable to spread the virus. The stylet bundle is composed of two maxillary stylets and two mandibular stylets contained in the labium [11]. When the mealybug does not feed, the stylet bundle is retracted as a loop in the body cavity of the insect inside a sheath called the crumena [12]. There is no evidence that the maxillary stylet tips in mealybugs contain the acrostyle region found to be associated with virus retention and transmission in aphids [13]. The food canal is connected to the foregut, comprising the precibarium, the cibarium, equipped with a muscular pump, and the esophagus [11]. During feeding, mealybugs extend the stylets into plant vascular tissues where the ingestion of primarily phloem begins, although xylem-sap feeding has also been documented through electropenetrography [14]. GLRaV-3 is phloem-restricted in both *Vitis vinifera* and *Nicotiana benthamiana;* thus, mealybug vectors must ingest phloem-sap to acquire virions for subsequent transmission [15].

The mechanisms of virus–vector interactions of GLRaV-3 in mealybugs are poorly understood. In this work, we used a membrane feeding system to show that GLRaV-3 can be transmitted from purified preparations of *V. vinifera* or *N. benthamiana*. Using an immunofluorescence approach, virions were localized to two retention sites in *P. ficus* mouthparts. We also tested molecules capable of disrupting virus transmission to gain a general idea of virus–vector interactions. We were successfully able to block GLRaV-3-transmission in *P. ficus*, demonstrating this approach is valid and can be used to screen other candidate blocking molecules.

## 2. Materials and Methods

### 2.1. Membrane Feeding Transmission Assays

*P. ficus* colonies were maintained on butternut squash (*Cucurbita moschata*) at 22 °C, with a 16:8-h photoperiod. First instars were used for all experiments because they were shown to be the most efficient vector of GLRaV-3 [9]. GLRaV-3 virions (accession LR101; variant I) were purified as previously described [15,16]. Whole virion integrity from purifications was confirmed previously [15]. Three resuspended virion purification aliquots from either *N. benthamiana* expressing the turnip mosaic virus P1/HC-Pro or *V. vinifera* were immediately pooled and added to 1.8 mL of an artificial diet composed of 15% sucrose and 1% BSA in TE, as described previously [6]. Transgenic tobacco expressing P1/HC-Pro, an antiviral silencing suppressor, were used because transmission efficiencies using wildtype tobacco were very low, and previously published studies showed increased numbers of P1/HC-Pro *N. benthamiana* plants became infected when compared to wildtype *N. benthamiana* [15]. An artificial diet without the addition of purified virions was used as a control. Diets were placed in small glass dishes. Approximately 20 mealybugs were placed in 14- by 20-mm (diameter by height) plexiglass feeding chambers, and the opening was covered by a layer of thinly stretched parafilm. The feeding chamber was then placed, parafilm side down, into a small dish containing either artificial diet with GLRaV-3 virions or just artificial diet and covered to prevent light disruption. Mealybugs were given an acquisition access period (AAP) of 24 h on each respective diet before being manually removed from the feeding chamber with a small paintbrush and moved to healthy test *N. benthamiana* or *V. vinifera* (cv. Cabernet Sauvignon) (*n* = 10 mealybugs per plant). After a 4-day inoculation access period (IAP), mealybugs were manually removed from the test plant, and plants were treated with pesticides and moved to the greenhouse. Petiole samples were collected from all plants 4 months post-inoculation, and RNA extractions were completed on 100 mg of petiole tissue [17]. One-step reverse transcription–polymerase chain reaction (RT–PCR) was then performed, and PCR products were analyzed using fragment analysis as described previously [17]. The primers used were Fwd 5′- NED-AAG TGC TCT AGT TAA GGT CAG GAG TGA-3′ and Rev 5′- GTA TTG GAC TAC CTT TCG GGA AAA T-3′. Logistic regression analysis was performed using R (Version 3.0.2, R. RStudio, Inc., Boston, MA, USA [http://www.rstudio.com/]).

### 2.2. Virion Localization Assays

Potential virion retention sites in mealybug vectors were localized following the protocol previously described [6]. For artificial diet experiments, approximately 100 first instar mealybugs at a time were placed in feeding chambers as described above and placed on artificial diets with or without GLRaV-3 virions for a 12-h AAP. This assay was repeated for 14 biologic replicates. In a second experiment, mealybugs were placed on GLRaV-3 source or healthy vine cuttings (accession LR101; variant I) for a 12-h AAP (3 biologic replicates). A 12-h AAP was used in localization assays after we determined that AAPs longer than 12 h resulted in lower labeling efficiencies (CAP personal observations). Mealybugs were then placed on a second diet containing a 1/800 dilution of rabbit anti-GLRV-3 polyclonal antisera (kindly supplied by Dr. Adib Rowhani, UC Davis) for 12 h followed by a third artificial diet containing a 1/200 dilution of goat anti-rabbit antisera conjugated with Alexa Fluor 488 (Invitrogen, Carlsbad, USA) for 12 more hours. A final artificial diet was presented to the mealybugs for 4 h as a wash to remove any nonspecifically bound virions or leftover antibodies present in the mouthparts. Mealybugs were placed on a glass slide in a drop of glycerol, covered with a coverslip and observed using a Zeiss Axio Imager fluorescence microscope using a YFP filter set (Chroma Filter Set #49,003) to minimize mealybug autofluorescence (excitation 490–510 nm, dichroic 515 nm, emission filter 520–550 nm). Images were captured using a QIClick digital CCD grayscale camera. The biologic replicates were combined, and logistic regression analysis was performed using R.

### 2.3. Virus Transmission

Five to ten mealybugs were placed in feeding chambers, and the opening was covered by a layer of thinly stretched parafilm as described above. The feeding chambers were then placed, parafilm side down, into a small dish containing either artificial diet with a competitor molecule or just artificial diet for a 12-h AAP. The competitor molecules chosen were the lectin wheat germ agglutinin (0.1% *v/v*) with expected affinity to substrates on the cuticle of insect vectors [18] and casein (0.1% *v/v*), a molecule commonly used to block nonspecific binding of proteins. Mealybugs were then moved to an artificial diet containing viral purifications from grapevines as described above for another 12 h, followed by manual placement onto test *V. vinifera* for a 4-day IAP. Petiole samples were collected from plants 4 months post-inoculation, and RNA extractions and RT–PCR were completed as described above. Logistic regression with Firth’s bias correction was used because no acquisition or inoculation occurred in the sucrose treatment resulting in all zeroes (quasi-complete separation of factor levels) [19]. Analyses were performed using R and the logistf package for Firth’s logistic regression [20].

## 3. Results

### 3.1. Mealybug Transmission of GLRaV-3 through an Artificial Diet Membrane System

To determine if *P. ficus* could transmit GLRaV-3 from artificial diets to test plants, experiments with virus purified from both *V. vinifera* and P1/HC-Pro *N. benthamiana* were conducted (Table 1). Our results indicate that *P. ficus* feeds on solutions from both purified GLRaV-3 plant sources through parafilm membranes and transmits the virus to *V. vinifera* and P1/HC-Pro *N. benthamiana*; *P. ficus* transmitted GLRaV-3 from *V. vinifera* virus purifications to 12 out of 94 *V. vinifera*, and only 1 out of 187 P1/HC-Pro *N. benthamiana* tested four months post-inoculation; *P*. *ficus* specimens fed on diets containing P1/HC-Pro *N. benthamiana* virus-purifications transmitted GLRaV-3 to 1 out of 84 *V. vinifera* and 2 out of 125 P1/HC-Pro *N. benthamiana* recipient plants. The main effects of source (*p* = 0.368, z = −0.900) or recipient (*p* = 0.808, z = −0.243) plants did not have a significant effect on transmission rates. The interaction within the same plant species transmission was significant when compared to interactions between the two different species (*p* = 0.026, z = 2.226). All trials, including *P. ficus* feeding on artificial diet without purified virus as controls, resulted in no transmission.

### 3.2. GLRaV-3 Virions Are Retained in the Mouthparts of Vectors

**Figure 1:**
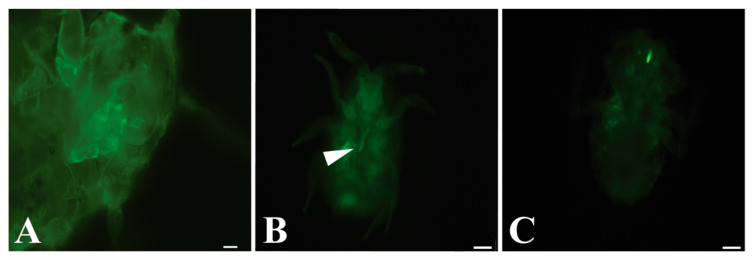
GLRaV-3 virions are retained in the mouthparts of *P. ficus* vectors. (**A**) First instar head of *P. ficus* control after feeding on membrane diet containing sucrose without virions followed by subsequent antibody labeled diets showing no labeling. Chitin in insects can be autofluorescent, accounting for the small signal observed. (**B**) Retention of GLRaV-3 virions in the retracted stylet tips (near white arrow) or (**C**) cibarium of *P. ficus* after sequential membrane feeding immunolocalization assay. Bars represent 20 µm.

*P. ficus* were sequentially fed on separate artificial liquid diets containing first, purified GLRaV-3 virions, second, anti-GLRaV-3 IgG, and third, a diet with goat anti-rabbit IgG conjugated with Alexa Fluor 488. A final short wash step on artificial diet alone was included to reduce any nonspecific binding. The same experiment was repeated, placing *P. ficus* on GLRaV-3-infected grapevines instead of viral purifications during the first step, followed by subsequent diets as described above. All trials included *P. ficus* feeding on artificial diet without purified virus or on healthy plants as controls. No fluorescent labeling was observed in the control experiments. Our results show that GLRaV-3 was retained in two binding sites in insect mouthparts (Figure 1). When feeding on purified virions, 19 out of the 645 specimens of *P. ficus* showed a fluorescent signal in the tip of stylets retracted in the labium (Figure 1b, Figure S1). Fluorescent signal was observed in the cibarium of 11 individuals (Figure 1c); in one insect signal was observed in both the cibarium and stylet (Table 2). No labeling was observed in any of the 149 insects fed on artificial diet without virions. When *P. ficus* was provided with GLRaV-3-infected plants instead of purified virions, four insects were observed with a fluorescent signal in the tip of stylets, and four insects showed labeling in the cibarium out of 298 individuals observed. No labeling was observed in any of the 35 mealybugs fed on healthy plants. There was no significant effect of the diet (artificial or cuttings) (*p* = 0.808, z = −0.242) on the location of the signal observed, and there was no significant difference in whether the signal was located in the stylet or cibarium (*p* = 0.190, z = 1.311) of insects observed.

### 3.3. GLRaV-3-Transmission Is Blocked by a Lectin

Because GLRaV-3 appears to be retained in one or two binding sites, further questions remain regarding the nature of the receptors involved in the mouthparts. Virus transmission experiments were conducted by providing a blocking compound to *P. ficus* in artificial diets, followed by feeding on purified virus and subsequent inoculation on *V. vinifera* (three biologic replicates) to determine if transmission could be disrupted. The competitor molecules tested were the lectin wheat germ agglutinin (WGA) with expected affinity to substrates on the cuticle of insect vectors and casein, a molecule commonly used to block nonspecific binding of proteins in immunoassays. Our results show that WGA significantly blocked GLRaV-3-vector transmission to plants (0/45 tested) in comparison to the sucrose controls (9/46) (Table 3; X^2^ = 10.492, *p* = 0.001). Four of 45 plants were GLRaV-3-positive when blocked with casein, with no significant difference when compared to sucrose controls (9/46) (X^2^ = 1.963, *p* = 0.161). As a control, experiments with *P. ficus* fed on sucrose instead of a blocking molecule resulted in 9 of 46 plants infected with GLRaV-3. This provides evidence that the virus binds to *P. ficus* mouthparts and suggests that there is a receptor implicated in virus retention. This assay also provides proof of concept that this approach is valid for testing other compounds capable of blocking transmission.

## 4. Discussion

GLRaV-3 is one of the most important viruses of grapevines, but there are significant gaps in our understanding of its transmission biology. GLRaV-3 research has been limited by a labor-intensive and technically challenging host–pathogen system until the recent discovery that GLRaV-3 is capable of infecting an alternative model host, *N. benthamiana* [15]. Building off that study, we determined that GLRaV-3 purifications from both P1/HC-Pro *N. benthamiana* and *V. vinifera* could be transmitted through an artificial diet membrane system to healthy plants. The purification protocol used was based on a protocol previously described for LIYV, a related long filamentous virus [16]. As determined in previous work, we eliminated subsequent steps associated with ultra-pure purifications to preserve the integrity of the long flexuous virions as well as maintain virus yields [15].

GLRaV-3-transmission was highest from *V. vinifera* purifications to *V. vinifera* plants compared to transmission to P1/HC-Pro *N. benthamiana* or from P1/HC-Pro *N. benthamiana* purifications to *V. vinifera* or P1/HC-Pro *N. benthamiana* plants. It is possible that transmission could be affected by *P. ficus* host plant preference. Observations of *P. ficus* behavior on *V. vinifera* versus *N. benthamiana* have been previously described and demonstrated that *P. ficus* prefers *V. vinifera* [15]. In these experiments, it was thought that the membrane feeding would eliminate the plant preference component affecting transmission results, but that was not observed given the low transmission rate from *N. benthamiana* purifications; *P*. ficus may reject *N. benthamiana* as a recipient host, or there is some component purified from *N. benthamiana* that deters *P. ficus* feeding on diets. It also may be that *P. ficus* prefers probing plants instead of parafilm, as the transmission rates published in earlier work from *N. benthamiana* or *V. vinifera* plants to both *N. benthamiana* and *V. vinifera* plants were higher [15]. Lastly, virus particle integrity is affected in preparations compared to plant hosts. Future studies should compare GLRaV-3-transmission from purifications with other mealybug species that may feed on *N. benthamiana*. Furthermore, while this work demonstrates that artificial diets may be used to study mealybug–ampelovirus interactions and that the protocol used for virus purification yields intact particles, methodological improvements would allow for a range of additional hypotheses to be tested.

Because *P. ficus* transmitted GLRaV-3 from artificial diets through membrane feeding, a unique immunofluorescent localization system previously used to investigate LIYV transmission in whitefly vectors was adapted for this study. This approach proved to have low efficiency in this host–pathogen system compared to the results observed in whiteflies, with 31 out of 645 first-instar *P. ficus* observed with a fluorescent signal (Table 2). A similar pattern was observed in 8 out of 298 *P. ficus* fed on GLRaV-3-infected source grapevine cuttings instead of viral purifications (Table 2). In order to observe fluorescence, *P. ficus* was required to feed on four different subsequent diets. If the mealybug did not feed on any one of the diets, specific labeling would not be observed, which could explain the low numbers of insects with any labeling. We observed fluorescent signals in the anterior foregut region (cibarium) of *P. ficus* as well as on the retracted stylet tips, regardless of the initial source diet (viral purifications or live plant cuttings). In one insect, a signal was observed in both of these regions at the same time. It is possible that the virus is retained in both sites, but further studies testing other GLRaV-3 variants and mealybug vector species should confirm if transmission of the virus is associated with one or both of the sites. Without associated transmission data, it cannot be concluded whether the retention sites observed are implicated in the transmission of GLRaV-3.

Although it cannot be confirmed which site is associated with transmission from this work, it is encouraging that these results follow trends observed in other virus–vector systems. Both the stylet tips and cibarium regions have been implicated in nonpersistent or semipersistent virus transmission. The retention site of the related noncirculative LIYV has recently been identified in the cibarium of the whitefly vector [6]. Retention sites for other semipersistently transmitted viruses including the leafhopper-transmitted *Maize chlorotic dwarf virus*, aphid-transmitted *Anthriscus yellows virus*, and *Parsnip yellow fleck virus* have also been localized to the tips of stylets or foreguts of insect vectors [21,22,23]. The stylet tips of aphid vectors were observed to be the retention site for CaMV, another semipersistent virus, as well as *Cucumber mosaic virus* and other potyviruses [1,24].

In an effort to further characterize the nature of virus-vector interactions, we tested if the transmission of GLRaV-3 could be disrupted by either casein or the lectin wheat germ agglutinin (WGA) [25]. Our results showed that WGA, a lectin with affinity to substrates on the cuticular surface of insect vectors, resulted in significantly lower transmission rates than casein, a molecule used to block nonspecific binding of proteins or the sucrose control. WGA is known to bind to chitin, the long-chain polymer of N-acetylglucosamine, as well as sialic acid, which has been shown to be a receptor for some animal viruses [26]. In *Citrus tristeza virus*, a member of the Closteroviridae family, virions were shown to bind to the *N*-acetylglucosamine moieties of the cuticular surface of the aphid cibarium [7]. Further studies are needed to determine whether WGA is blocking GLRaV-3 from binding to N-acetylglucosaminated proteins or sialylated proteins in mealybug mouthparts. These results demonstrate that our approach to feed mealybugs blocking molecules and determine transmission rates is valid and that it is feasible to test other molecules for this purpose. Previous work showed that lectins, carbohydrates, antibodies, and peptides affected the transmission rate of *Xylella fastidiosa*, a noncirculative bacterial pathogen that colonizes the foregut of leafhopper vectors [18,25]. This approach provides only a general idea of the type of vector–pathogen interactions. For vector-borne plant viruses, a specific viral protein is required for virus transmission, and these have been recently described for related viruses in the family Closteroviridae. The minor coat protein (CPm) for the *Crinivirus* LIYV and both the CPm and heat shock proteins in *Citrus tristeza* virus, an aphid-transmitted *Closterovirus*, are viral proteins required for successful retention and transmission by insect vectors [6,7]. Further research investigating the interruption of transmission processes is required to develop novel control strategies as well as develop a basic understanding of transmission mechanisms [27].

In conclusion, our analyses of GLRaV-3-transmission mechanisms suggest that viral retention can be narrowed down to one or two binding sites in *P. ficus* mouthparts. Although the viral proteins required for binding remain unknown, vector transmission was blocked after binding of WGA, suggesting that the virus interacts with the cuticular surface of the mouthparts. This provides first insights into the transmission biology of this economically important host–pathogen system and demonstrates that mealybug feeding through artificial diet systems works for future studies. Further investigations are needed to elucidate the specific viral proteins required for transmission. The creation of recombinant viral proteins and specific antibodies could help confirm the viral retention site and transmission strategies of this system.

## Figures and Tables

**Table 1:** *Planococcus ficus* transmits GLRaV-3 from purified virus fed through a membrane system. GLRaV-3 was purified from both *Vitis vinifera* and P1/HC-Pro *Nicotiana benthamiana* source plants and successfully transmitted by insect vectors feeding on artificial diets with an acquisition access period of 24 h followed by a four-day inoculation access period on test plants. All trials included *P. ficus* feeding on artificial diet without purified virus as controls.

Source	Recipient	Plants Infected/Plants Inoculated	Control
*V. vinifera* diet	*V. vinifera*	12/94	0/18
	*N. benthamiana*	1/187	0/37
*N. benthamiana* diet	*V. vinifera*	1/84	0/17
	*N. benthamiana*	2/125	0/25

**Table 2:** Summary of GLRaV-3 retention site observations. *Planococcus ficus* fed on either artificial diet augmented with GLRaV-3 virions or vine cuttings for 12 h, followed by 12-h acquisition access times on diets containing anti-GLRV-3 polyclonal antisera and antisera conjugated with Alexa Fluor 488, respectively.

Source		Stylet	Cibarium	Both	Total
Artificial diet	Number of *P. ficus* labeled	19	11	1	31
	*P. ficus* controls ^*^	0	0	0	149
	Total *P. ficus* viewed				794
GLRaV-3 vine cuttings	Number of *P. ficus* labeled	4	4	0	8
	*P. ficus* controls ^†^	0	0	0	35
	Total *P. ficus* viewed				333

^*^P. ficus fed on artificial diet without purified GLRaV-3 virions. ^†^ *P. ficus* fed on vine cuttings from healthy *V. vinifera*.

**Table 3:** GLRaV-3-transmission is reduced by a lectin. (**a**) Results from three biologic replicates of GLRaV-3 blocking transmission tests showing the number of plants infected/plants inoculated. (**b**) Statistical results from bias-corrected logistic regression testing differences between wheat germ agglutinin (WGA) and casein transmission results from sucrose controls.

(**a**)					
**Treatment**	**1**	**2**		**3**	**Total Positive**
WGA	0/15	0/15		0/15	0/45
Casein	1/15	1/15		2/15	4/45
Sucrose	4/16	3/15		2/15	9/46
(**b**)					
**Treatment**	**Estimate**		**SE**	**χ^2^ Statistic**	***P*** **Value**
Intercept	−1.204		0.517	6.331	0.011
Casein	−0.828		0.608	1.963	0.161
WGA	−3.103		1.429	10.492	0.001

## Supplementary Materials

The following are available online at https://www.mdpi.com/1999-4915/12/8/843/s1, Figure S1: Evidence of GLRaV-3 virion retention in the stylet tip of *P. ficus* vectors.
