# Supplementary figures and images for: A Lectin Disrupts Vector Transmission of a Grapevine Ampelovirus
Source: Viruses. 2020 Aug 1;12(8):843. doi: 10.3390/v12080843 (PMC7472352; doi:10.3390/v12080843)

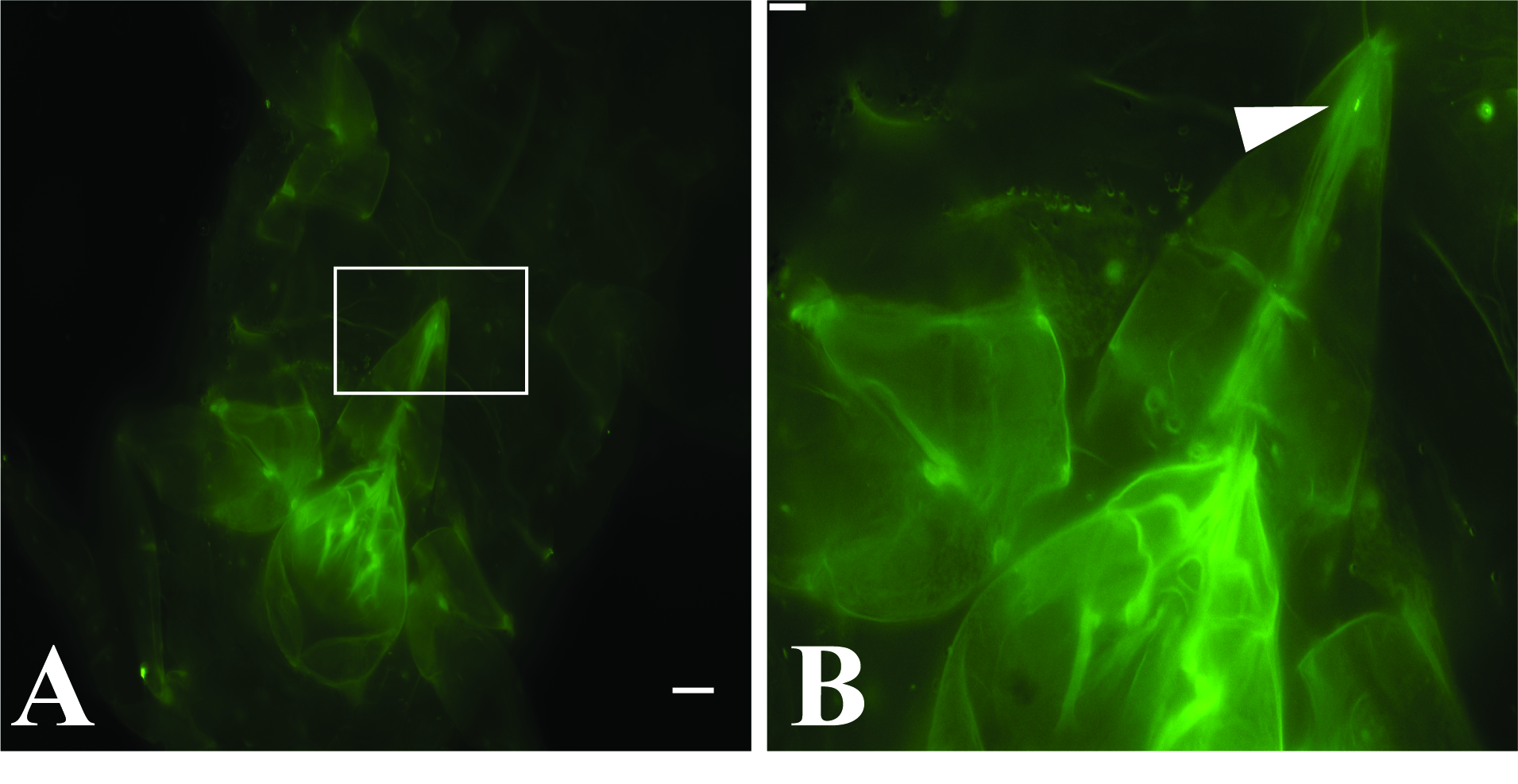

Supplement: Supplementary file 1 [file viruses-12-00843-s001.tif]
